# *Mycobacterium chelonae* Skin Infection in Kidney-Pancreas Recipient

**DOI:** 10.3201/eid1102.040902

**Published:** 2005-02

**Authors:** Ingrid Stelzmueller, Karin M. Dunst, Silke Wiesmayr, Robert Zangerle, Paul Hengster, Hugo Bonatti

**Affiliations:** * Innsbruck Medical University, Innsbruck, Austria

**Keywords:** kidney transplant, pancreas transplant, Mycobacterium chelonae, mycobacteria

**To the Editor:** *Mycobacterium chelonae* is rapid growing and is ubiquitous in the environment, including soil, water, domestic and wild animals, and milk and fruit products. It can be associated with infections of the soft tissue, lung, bone, joint, central nervous system, and eye. *M. chelonae* infections in an immunocompromised host are disseminated in >50% of those infected; chronic use of steroids, even in low doses, seems to be the most important predictive factor for disseminated disease (*1**,**2*). In immunocompetent hosts, nontuberculous mycobacteria can colonize body surfaces and be secreted for prolonged periods without causing disease. In hematopoietic stem cell and solid organ transplant recipients, infections with nontuberculous mycobacteria are common and may be a source of illness and death (*3*). We describe a case of localized cutaneous *M. chelonae* infection after a dog bite in a kidney-pancreas transplant recipient.

A 43-year-old female patient underwent kidney transplantation for diabetic nephropathy in 1985. After loss of organ function due to chronic rejection, she underwent combined kidney-pancreas transplantation 5 years later, in 1990. Because of chronic rejection, the patient lost the kidney graft 5 years later, in 1995, and went back on dialysis with a well-functioning pancreas graft. In 2004, the patient was bitten on the right forearm by a dog. She was on immunosuppressive therapy of prednisolone (5 mg/day), cyclosporine-A (trough levels of 100 ng/dL), and azathioprine (50 mg/day). The initial lesion healed without major complication. After several days, a single firm edematous plaque of 3 x 5 cm developed at the site of the animal bite, and the patient was admitted to the Department of Dermatology. Empiric antimicrobial combination therapy, including clindamycin (300 mg every 8 hours) and ciprofloxacin (500 mg every 12 hours), was initiated. As no clinical improvement was achieved, a biopsy was performed, which showed a granulomatous inflammation with a high number of mycobacteria (Figure). Atypical mycobacteria were cultured from a second biopsy (Löwenstein-Jensen/Stonebrink, Heidelberg, Germany); *M. chelonae* was identified by polymerase chain reaction. Therefore, antimicrobial therapy was changed to clarithromycin (500 mg twice daily) for 6 months. Although cyclosporine-A dosage was reduced with initiation of antimicrobial therapy, trough level increased to 350 ng/mL; therefore, further dose reduction was performed. Within a few weeks, the lesion disappeared completely, and the patient retained good pancreatic graft function. To rule out dissemination to other organs, a computed tomography of head, thorax, and abdomen was performed at time of diagnosis.

**Figure:**
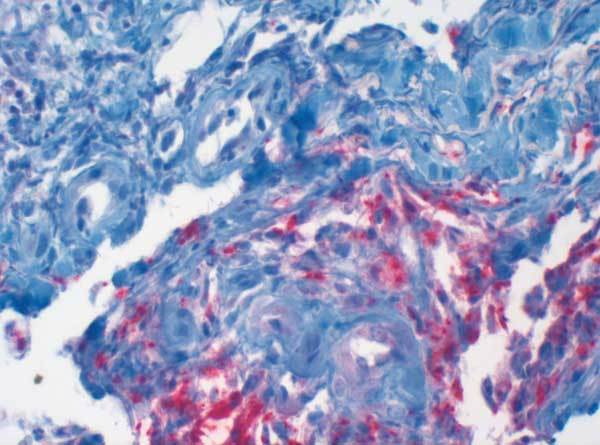
Skin biopsy of the lesion showed granulomatous infection with *Mycobacterium chelonae* (Ziehl-Neelsen stain, x40).

In the immunocompromised host, an uncontrolled proliferation of primarily colonizing or contaminating pathogens or commensals can progress to severe disease. Diagnosis is often difficult because patients with these infections may have atypical symptoms due to immunosuppressive therapy. If diagnosis is made early, dissemination can likely be avoided. Therefore, suspicious cutaneous lesions should be biopsied for histopathologic examination, and special stains and tissue cultures should be performed for detecting fungi, viruses, and bacteria, including mycobacteria (*3**,**4*). Nontuberculous mycobacteria are resistant to conventional tuberculostatic therapy and have variable susceptibility to other antimicrobial agents (*1**,**2**,**5*). Clarithromycin seems to be the most active drug, and azithromycin might also have good activity (*3**–**6*). Clarithromycin has been administered successfully as monotherapy, and our case confirms these data. However, several cases of resistance have been described, and use of at least 1 other drug, such as an aminoglycoside or a quinolone, in addition to clarithromycin has been recommended (*1**,**4**,**7**–**9*). Clarithromycin is a potent inhibitor of cytochrome P450 (*3**,**4*). Therefore, cyclosporine-A and tacrolimus levels have to be monitored exactly, and dose adjustments may be required. Duration of therapy depends on the isolate, site of infection, and clinical response to therapy, but in general, it should be continued for at least 6 months (*3**,**8*).

Thus far only a few cases of infections with *M. chelonae* in kidney, heart, liver, and lung transplant recipients have been described (*3*). Most of these infections were disseminated and often resulted in chronic infection. To our knowledge, this report is the first of localized cutaneous disease from *M. chelonae*, which completely healed within 3 months, in a kidney-pancreas transplant recipient. Although *M. chelonae* might be part of the colonizing oral flora of dogs, it is more likely that the bite contributed to translocation of the transient dermal flora. Any factor that disrupts the skin barrier, such as insulin self-injection in diabetes patients, surgical wound, insect sting, or animal bite, might be associated with this type of infection (*10**,**11*). We conclude that early diagnosis prevents dissemination, leads to rapid clinical response, and allows antimicrobial monotherapy with a macrolide. Such an approach preserved the function of the pancreatic allograft.
